# A High-Performance Food Package Material Prepared by the Synergistic Crosslinking of Gelatin with Polyphenol–Titanium Complexes

**DOI:** 10.3390/antiox13020167

**Published:** 2024-01-29

**Authors:** Wanqin Zhang, Jiaman Liu, Tao Zhang, Bo Teng

**Affiliations:** 1 College of Science, Shantou University, Shantou 515063, China; 22wqzhang@stu.edu.cn (W.Z.); 19jmliu1@stu.edu.cn (J.L.); 2 Key Laboratory of Marine Materials and Related Technologies, Ningbo Institute of Materials Technology and Engineering, Chinese Academy of Sciences, Ningbo 315201, China; 3 Guangdong Provincial Key Laboratory of Marine Biotechnology, Shantou University, Shantou 515063, China

**Keywords:** gelatin film, polyphenol–titanium complexes, synergistic effect

## Abstract

This study aims to enhance gelatin film performance in the food industry by incorporating polyphenol–titanium complexes (PTCs) as crosslinkers. PTCs introduce multiple linkages with gelatin, including coordination and hydrogen bonds, resulting in synergistic crosslinking effects. This leads to an increased hydrodynamic volume, particle size, and thermal stability of the gelatin films. Compared to films crosslinked solely by polyphenols or titanium, PTC-crosslinked gelatin films exhibit significant improvements. They show enhanced mechanical properties with a tensile strength that is 1.7 to 2.6 times higher than neat gelatin films. Moreover, these films effectively shield UV light (from 82% to 99%), providing better protection for light-sensitive food ingredients and preserving lutein content (from 74.2% to 78.1%) under light exposure. The incorporation of PTCs also improves film hydrophobicity, as indicated by water contact angles ranging from 115.3° to 131.9° and a water solubility ranging from 31.5% to 33.6%. Additionally, PTC-enhanced films demonstrate a superior antioxidant ability, with a prolonged polyphenol release (up to 18 days in immersed water) and a higher free radical scavenging ability (from 22% to 25.2%). Overall, the improved characteristics of gelatin films enabled by PTCs enhance their performance, making them suitable for various food packaging applications.

## 1. Introduction

Packaging is essential for food preservation, as it can maintain food quality and extend the shelf life of food products. Packages have been dependent on fossil fuel-derived materials for a long time, such as polyethylene terephthalate (PET), polypropylene (PP), and polystyrene (PS) [1]. However, due to the environmental issues associated with plastics and the uncertainties in the global oil market, how to reduce the use of plastic packaging has become a pressing concern. Since then, natural materials have become a new choice for the food industry. But the physical and barrier properties of natural materials need to be improved before more industrial applications are devised [2].

Collagen is an protein with a triple-helix structure. Collagen comprises peptide chains mainly consisting of glycine-proline-hydroxyproline sequences [3]. Collagen is rich in terrestrial and aquatic animal tissues, such as skins, hides, tendons, cartilage, bones, and scales [4]. These tissues are being treated as by-products or even solid wastes by the meat and fish industries. Gelatin, also known as partially hydrolyzed collagen, is a promising film material with renewable properties that can be applied onto food surfaces to prevent bacterial contamination [5]. But the native gelatin exhibit hydrophilic characteristics, and the films have limited physical properties, which cannot fulfill the requirement of packaging semi-moist or moist foods [2].

Crosslinkers are responsible for forming additional bonds and forces within the gelatin molecules. They can strengthen the three-dimensional gelatin networks, enhancing the physical properties of gelatin films. Polyphenols are secondary metabolites in vascular plants that normally function as antioxidants in our daily diet. Recently, polyphenols have been extensively reported as fine crosslinkers because they can provide additional functions, such as a UV-light-absorption ability [6,7,8] and anti-bacterial properties [9] for the films. The polyphenols from various kinds of plants, such as green tea [6], pomegranates [10], and lingonberries [11], have been reported to improve the mechanical properties and antioxidant capabilities of films. Furthermore, several monomers, including catechin [11], epicatechin [12], epigallocatechin gallate [13], and gallic acid [14], have also been reported as fine crosslinkers. These monomers can also improve the performance of gelatin films in terms of having better mechanical properties, improved light shielding, and antioxidant and antimicrobial abilities.

It is worth mentioning that polyphenols have catechol or pyrogallol moieties on their molecules, allowing them to form polyphenol–metal complexes via chelation with metal ions [15,16]. These complexes have been extensively studied as versatile platforms for creating advanced materials, including nanoparticles, nanocapsules, and surface coatings [17]. However, there is a noticeable shortage of studies focusing on the application of metal–polyphenol complexes in edible films.

Herein, three kinds of polyphenol–titanium complexes (PTCs), including polyphenol polymer–titanium complexes (PPTCs), polyphenol dimer–titanium complexes (PDTCs), and polyphenol monomer–titanium complexes (PMTCs), were incorporated into gelatin films as crosslinkers. Subsequently, the underlying mechanism of the crosslinking process was studied. The properties of the films, including physical properties, light shielding capabilities, water solubilities, and antioxidative capacities, were evaluated. Notably, the properties of the films showed remarkable improvement after being crosslinked by the PTCs. Their tensile strength increased significantly (up to 47.55 MPa, which is 2.6 times higher than that of neat gelatin film), the UV light shielding reached up to 1% light transmission from 200 to 400 nm, the protection of light-sensitive food ingredients achieved up to 78.1% lutein retention after 10 h of strong light exposure, the water contact angle increased to up to 131.9°, water solubility reduced to less than 33.6%, and antioxidant activity improved (up to 25.2% ABTS scavenging ability after 18 days of water immersion).

## 2. Materials and Methods

### 2.1. Materials

The following materials were obtained from Aladdin Biochemical Technology Co., Ltd. (Shanghai, China): glycerol, sodium carbonate, ammonium acetate, 2,2′-azino-bis (3-ethylbenzthiazoline-6-sulfonic acid (ABTS), K_2_S_2_O_8_, and ethanol. Lutein and gelatin (from pork skin, with a bloom value of 300 g) were purchased from Sigma Aldrich (Shanghai, China). Hops were harvested from Jinhua farm (100°47′75.5″ E, 38°96′95.7″ N) from Gansu province, China.

**Figure 1:**
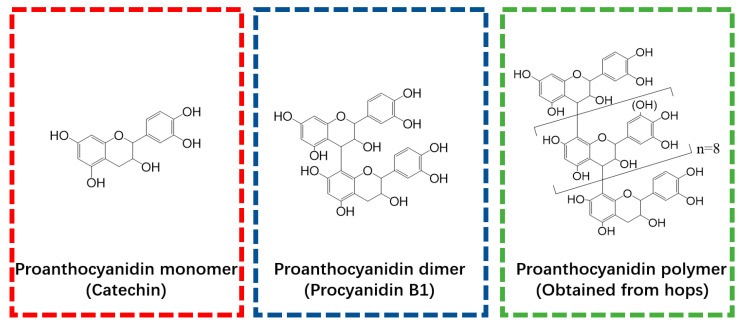
Molecular structures of polyphenol monomer, dimer, and polymer that were employed in the present study.

Catechin, procyanidin B_2_, and Ti(SO_4_)_2_ were obtained from Sigma Aldrich (Shanghai, China) and used as polyphenol monomer (PM), polyphenol dimer (PD), and titanium (T), respectively. The polyphenol polymer (PP) used in this study was extracted from hops. The methods used for PP extraction, purification, and structural characterization have been reported in our previously published paper [18] and are also shown in Protocol S1. The structure of the PP was characterized by HPLC-ESI-MS/MS and ^13^CNMR, and the results are provided in Figures S1–S3 and Table S1. To illustrate the polyphenols used in this research, the structures of PM, PD, and PP are presented in Figure 1.

### 2.2. Analysis of the PTCs

#### 2.2.1. Preparation of PTCs

The polyphenols were, respectively, dissolved in water to prepare different polyphenol solutions with same concentration (0.25 mg/mL). Subsequently, a 1 mL solution of polyphenols was combined with a 1 mL Ti(SO_4_)_2_ solution (0.25 mg/mL) and vigorously mixed for 30 s via vortexing to prepare PMTCs, PDTCs, and PPTCs, respectively.

#### 2.2.2. UV-Vis Spectra of the PTC-Containing Solution

A total of 200 μL of the PTC solutions were taken and transferred to a quartz 96-well plate for UV-Vis analysis. The absorptions were recorded by a microplate reader (BioTek Synergy™ HTX, Winooski, VT, USA) within a wavelength range of 250–800 nm. Furthermore, spectra of the polyphenol solution (0.125 mg/mL) and Ti(SO_4_)_2_ solution (0.125 mg/mL) were also recorded to facilitate a comparison.

#### 2.2.3. Fluorescence Spectrum of the PTC-Containing Solution

The analysis was conducted following the methodology outlined in our previous report [18]. A 1 mL of PTCs solution was transferred into a quartz cuvette. The emission fluorescence were measured through 300–500 nm using a fluorescence spectrometer (Hitachi F-7000, Kyoto, Japan) with an 280 nm excitation wavelength.

#### 2.2.4. SEM Analysis of the PTCs

A total of 5 μL of the PTC solution was transferred on to a silicon slice and dried in the air. The slice was then fixed on a SEM specimen holder. The PTCs were subjected to gold coating in vacuum using an Eiko Sputter Coater at a 5 kV voltage. Surface characteristics of the PTCs were observed through a SEM instrument (Gemini 300 field emission, ZEISS, Oberkochen, Germany).

### 2.3. Observation of PTCs within Gelatin Films

#### 2.3.1. Film Fabrication

The gelatin films were fabricated employing a solution-based approach with slight adjustments based on the methodology outlined before [19]. A total of 4 g of gelatin was dissolved in 100 mL of water and subjected to stirring at a temperature of 80 °C for 1 h. To prevent drying induced film cracking, the mixture was then homogenized with 1 g of glycerol (~1% of the film casting solution) as plasticizing agent [19]. The Ti(SO_4_)_2_ solution and/or polyphenol solutions were subsequently added as crosslinkers to prepare the gelatin blends. The dosages of crosslinkers were chosen based on a previous report [19], and the composition of each gelatin blend is summarized in Table 1.

The mixture was homogenized through stirring (100 rpm) at 80 °C for 2 h. Afterwards, the pH of the blend was then adjusted to 7.0 by a Na_2_CO_3_ solution (0.5 mol/L). Then, 50 mL of the gelatin blend was transferred into a six-well Teflon plate (with dimensions of 10 × 5 × 1 cm for each well) and dried at 50 °C for 48 h to obtain the films. The films were analyzed before conditioning at 25 °C with 50% relative humidity for 48 h. Subsequently, the average film thickness was obtained by measuring at 12 random positions with a spiral micrometer (Table S2).

#### 2.3.2. X-ray Diffraction (XRD) Analysis

The structural characteristics of crystals formed within the gelatin films were tested through XRD analysis following the method outlined by Ellis and McGavin [20]. The diffraction signals were recorded from 4° to 50° (1°/min) using an X-ray diffractometer (D8 Advance, Bruker, Ettlingen, Germany) with a CuKα radiation source (λ = 1.5418A, 40 kV, and 40 mA).

### 2.4. Mechanism of Gelatin Crosslinking

#### 2.4.1. Attenuated Total Reflection Fourier Transform Infrared (ATR-FTIR) Analysis

The films were directly placed onto the pressure gauge on an IR spectrometer (Nicolet 200SXV, Thermo Fisher, Pleasanton, CA, USA), and the signals were recorded from 500 to 4000 cm^−1^ with a resolution of 2 cm^−1^. Each film underwent 50 scans, and air spectra were utilized for background correction. The spectra were further analyzed by the OMNIC™ Series software (version 9.2, Thermo Fisher, Pleasanton, CA, USA) to obtain the second-order derivative spectra [21].

#### 2.4.2. High-Performance Size-Exclusion Chromatography–Evaporative Light-Scattering Detector (HPSEC-ELSD) Analysis

Gelatin solutions were prepared by dissolving 400 mg of gelatin in a 100 mL of water at 80 °C. The solutions were subsequently mixed with or without polyphenol (8 mg) and Ti(SO_4_)_2_ (8 mg). After adjusting the resulting mixtures to pH 7.0 by adding the Na_2_CO_3_ solution (0.5 mol/L), the gelatin mixtures were then centrifuged for 30 min (10,000× *g*) to remove the precipitations. The liquid phases were then subjected to a RID-20A HPSEC-ELSD system (Shimadzu, Tokyo, Japan) for analysis. The analyses were carried out with reference to a previously published method [22], but modified as follows. For each analysis, a 20 µL sample was injected into consecutive TSK-GEL^®^ 3000 and 4000 columns (60 cm × 7.5 mm, 10 μm, column temperature = 50 °C) and eluted by an ammonium acetate solution (0.5 mol/L, dissolved in water, flow rate = 1.0 mL/min) as the mobile phase, the eluents were detected using an ELSD detector.

#### 2.4.3. Dynamic Light Scattering (DLS)

Gelatin mixtures were prepared as described in Section 2.4.2. Then, a 1 mL liquid phase of resulting mixture was transferred into a polystyrene cuvette and subjected to DLS analysis in accordance with Mo’s method [23]. The measurements were performed at 25 °C, with a 633 nm laser source and a detection angle of 90° using a Zetasizer instrument (nano ZS, Malvern, West Midlands, UK). For each sample, six replicates were performed, with each replicate comprising of 100 individual runs.

#### 2.4.4. Thermogravimetric Analysis (TGA)

In accordance with a previous report [24], 10 mg film were analyzed using 1100SF TGA 2 system (METTLER TOLEDO, Zurich, Switzerland), the changes in sample masses (see Figure S4) were monitored with spanning temperature from 25 to 550 °C (heating rate = 10 °C/min). Differential of the mass-temperature profiles were further calculated to obtain the derivative thermogravimetry (DTG).

### 2.5. Tensile Strength (TS) and Elongation at Break (EAB) of the Films

The TS and EAB were assessed following a published protocol [24]. The films were securely clamped onto a universal mechanical testing instrument (KY8600, Jinguan experimental instrument, Shanghai, China) to measure the maximum tensile force and length of film while reaching the breaking point. The film deforming process were applied with a 3 cm initial grip length, a loading cell with a capacity of 100 N, and a cross-head speed of 30 mm/min.

### 2.6. Light-Shielding Ability

#### 2.6.1. Light Transmission

The light transmission properties of the films were assessed using a published method [25]. The films were affixed to a clip attached to a UV/Vis Spectrometer (Lambda 950, PerkinElmer, Boston, MA, USA). Light transmittance was scanned from 200 nm to 800 nm, with a step size of 2 nm. To ensure accuracy, the transmittance spectrum was averaged over three replicates.

#### 2.6.2. Lutein Color-Fading Assay

The lutein color fading assay was performed in accordance with a previously published protocol [25], with modifications as described below. A 10 mL solution of lutein (4 mg/L) was placed into a glass bottle (1.5 × 1.5 × 10.0 cm^3^) that was wrapped with a single layer of gelatin film. The wrapped glass vial was then positioned at a distance of 30 cm from a light source system (CEL-HXUV 300, Zhong Jiao Jin Yuan Scientific Co., Ltd., Beijing, China), equipped with a 300 W Xe lamp (200–2500 nm) and an AM1.5 optical filter to remove infrared light. The samples (200 µL) were collected every 2 h from the beginning of light exposure, and the optical density at 446 nm was determined. The lutein retention was calculated by Equation (1):

Lutein retention (%) = 100 × (A_0_ − A_1_)/A_0_ (1)

where A_0_ and A_1_ were the optical density (at 446 nm) of sample before and after light exposure.

### 2.7. Surface Hydrophobicity and Water Solubility

#### 2.7.1. Water Contact Angle (WCA)

The sessile drop technique [26] was introduced for WCA analysis. The film was placed on an observing table attached to a CA100D contact angle tester (Topsun, Shanghai, China), then water was applied onto film surface with 5 μL of each droplet at a temperature of 298 K. Water contact process (at 0 s) was captured on video at a frame rate of 25 fps, and the water contact angle was determined by averaging 10 droplets for each film.

#### 2.7.2. Water Solubility (WS)

The WS was assessed using the procedure reported previously [27] and modified as follows: A 20 g of dried film sample was taken and immersed into a glass bottle with 200 mL of water. The glass bottle was then fixed into a shaking bath (30 rpm, 25 °C) and treated for 24 h. Afterwards, the liquid phase was removed by filtering through a filter paper, and the remaining solids were transferred to a 120 °C oven and heated for 6 h to obtain a constant weight.

### 2.8. Release of Polyphenols and Free Radical Scavenging Activities of the Films

#### 2.8.1. Antioxidant Activity of the Films

The films were solidified through immersing into liquid nitrogen within a mortar, followed by grinding with a pestle. Subsequently, 0.25 g of the ground powders was combined with 5 mL of methanol and vigorously stirred for 3 h. The resulting mixture underwent centrifugation at 5000× *g* for 30 min. The supernatant obtained from this process was subjected to analysis for DPPH radical scavenging activity, ABTS radical scavenging activity, and ferric reducing antioxidant power.

#### 2.8.2. DPPH Radical Scavenging Activity

The assessment of DPPH radical scavenging activity followed the methodology outlined by a previous report, with slight adjustments. A volume of 150 µL of the test sample was combined with 1.5 mL of 0.15 mM 2,2-diphenyl-1-picryl hydrazyl (DPPH) dissolved in 95% ethanol. The resulting mixture underwent vigorous mixing and was left undisturbed at room temperature in darkness for a duration of 30 min. Subsequently, the absorbance of the resultant solution was determined at 517 nm utilizing a spectrophotometer. A sample blank was prepared in an identical manner, with the exception that 95% methanol was substituted for the DPPH solution. Trolox was employed to establish a standard curve. The quantification of activity was calculated subsequent to subtracting the sample blank, and the results were expressed as micromoles of Trolox equivalents (TE) per gram of dried film.

#### 2.8.3. ABTS Radical Scavenging Activity

The assessment of ABTS radical scavenging activity adhered to the procedure established method. Stock solutions comprised a 7.4 mM ABTS solution (dissolved in 95% ethanol) and a 2.6 mM potassium persulphate solution. The working solution resulted from the equal combination of these stock solutions, followed by a 12 h reaction period at room temperature in darkness. Fresh ABTS solution was freshly prepared for each assay. The sample (150 µL) was combined with 2850 µL of ABTS solution, and the resulting mixture incubated at room temperature for 2 h in darkness. The absorbance was measured at 734 nm using a spectrophotometer. A sample blank was prepared in a similar manner, with the exception that methanol was utilized instead of the ABTS solution. Activity was calculated following the subtraction of the sample blank and expressed as micromoles of Trolox equivalents (TE) per gram of dried film.

#### 2.8.4. FRAP Radical Scavenging Activity

The FRAP assay was conducted following the protocol outlined before. The requisite stock solutions encompassed a 30 mM acetate buffer (pH 3.6), a 10 mM TPTZ (2,4,6-tripyridyl-s-triazine) solution in 40 mM HCl, and a 20 mM FeCl_3_ solution. A freshly prepared working solution was obtained by combining 25 mL of acetate buffer, 2.5 mL of TPTZ solution, and 2.5 mL of FeCl_3_ solution. This amalgamated solution underwent a 30 min incubation at 37 °C in a water bath and was denoted as the FRAP solution. For the assay, a sample volume of 150 µL was mixed with 2850 µL of the FRAP solution and left in darkness at room temperature for 30 min. The absorbance was measured at 593 nm using a spectrophotometer. A sample blank was prepared by excluding FeCl_3_ from the FRAP solution, and distilled water was employed as the replacement. Activity was determined after the subtraction of the sample blank and expressed as micromoles of Trolox equivalents (TE) per gram of dried film.

#### 2.8.5. Release of Polyphenols and Antioxidant Activities

Release of polyphenols from the gelatin films were examined by employing a prior study [13]. The maximum absorbance wavelength for polyphenols was determined in milli-Q water (monomer: *λ*_max_ = 280 nm; dimer: *λ*_max_ = 275 nm; polymers: *λ*_max_ = 282 nm). A film sample measuring 2 × 2 cm^2^ was placed into a 6 mL volume of water inside an amber glass bottle, which was subsequently positioned in a shaking bath (30 rpm, 25 °C). During each sampling interval, a volume of 200 µL was withdrawn from the liquid phase, and an equivalent volume of fresh water was added as a replacement. The light absorption of the liquid was measured with the wavelength set at the maximum absorbance of each polyphenol. The accumulated polyphenol release was calculated based on the light absorption of the liquid, with the light absorption of the immersed liquid from the gelatin film used as a reference for subtraction. The polyphenol release was then normalized by the polyphenol concentration in the initial film and expressed as a percentage. The published method [28] was employed to assess the ABTS radical scavenging activity as well.

### 2.9. Statisc Analysis of the Research

The data were expressed as mean of 3 replicates, the distinctions among the experimental samples were assessed using one-way analysis of variance (ANOVA) conducted with Minitab 17.1 (Minitab Inc., State College, PA, USA). In cases where the results exhibited significant differences (*p* < 0.05), Tukey’s posthoc test was employed to identify specific variations between the individual treatments.

## 3. Results and Discussion

### 3.1. Fabrication of PTC-Crosslinked Gelatin Films

**Figure 2:**
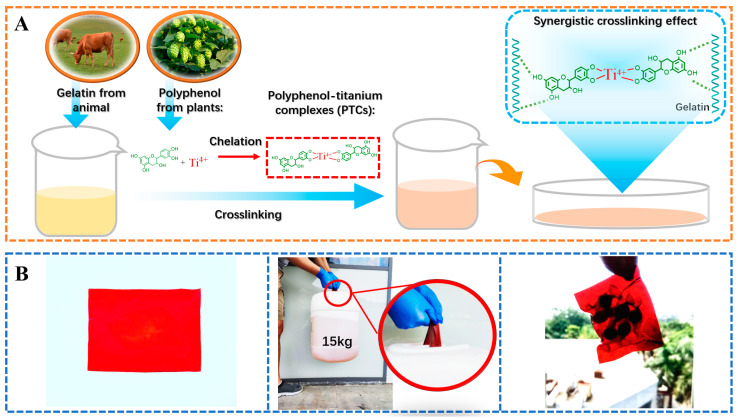
The crosslinking process of gelatin using PTCs (**A**), and the application of PTC-crosslinked gelatin films as a light-shielding material with improved mechanical properties for food packaging (**B**).

The present study explores the influence of incorporating polyphenols and titanium ions into crosslinking process to improve the performances of gelatin films. Theoretically, the titanium ions have the ability to interact with amide groups [21,29], while the hydroxyl groups in polyphenols can form hydrogen bonds with gelatin [11]. Therefore, the formation of PTCs within the gelatin films can potentially provide additional reactive sites, such as hydroxyl groups and titanium ions, which facilitate gelatin crosslinking (Figure 2A). These crosslinked gelatin films demonstrate impressive light-blocking properties and tensile strength (with an approximate thickness of 1.0 mm, capable of supporting a 15 kg weight). These characteristics render the films suitable for applications such as packaging bags and surface coatings (Figure 2B). The following sections summarize the evidence supporting the aforementioned hypotheses and provide detailed properties of the PTC-crosslinked films.

**Figure 3:**
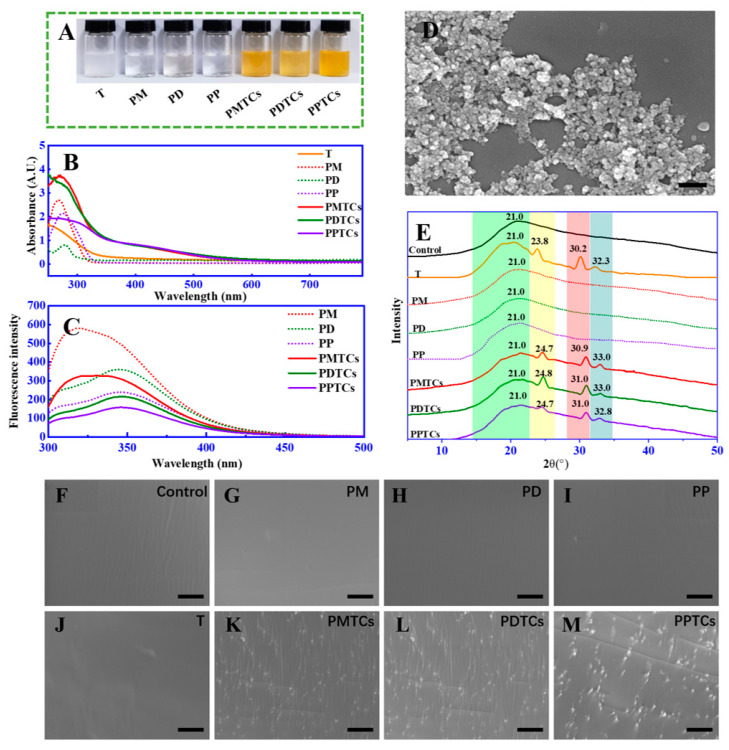
The color (**A**), UV-Vis spectra (**B**), fluorescence spectra (**C**), and SEM image (with scale bar = 200 nm) (**D**) of the PTCs; the XRD spectra (**E**) and surface morphology (with scale bar = 10 μm) (**F**–**M**) of the PTC-crosslinked gelatin films.

To validate the aforementioned theoretical hypothesis, we conducted experiments to investigate the formation of PTCs by directly reacting polyphenols (including monomers, dimers, and polymers) with titanium in an aqueous solution. After mixing, the color of the solutions immediately transitioned from colorless to orange (Figure 3A). New absorption peaks were observed in UV-Vis spectrum (320 to 550 nm) (Figure 3B). Additionally, significant fluorescence quenching of the polyphenols was observed across 320 to 350 nm (Figure 3C). Furthermore, SEM analysis identified the formation of nano-scaled particles within the size range of 80 to 100 nm (Figure 3D). Notably, both the changes in absorbances and fluorescence intensities displayed a positive correlation with the quantity of titanium added (Figure S4). These observations, such as the emergence of additional peaks within the visible range [15], color changes [30], fluorescence quenching [18], and the presence of particles [31], in agree with the characteristics of formation of metal–polyphenol complex and provide support for confirming the formation of PTCs in our current study.

The formation of PTCs within the gelatin networks was further assessed by XRD and SEM analyses. The XRD analysis (Figure 3E) of the neat gelatin film (control) revealed a peak at 2θ = 21.0°, which was contributed by the amorphous crystalline structure of the peptide chains [32]. The film crosslinked by titanium ions (T) exhibited additional diffraction peaks at 2θ = 23.8°, 30.2°, and 32.3°. According to Meagher’s report [32] and a standard XRD reference card (PDF 99-0020), these peaks can be attributed to the (210), (211), and (020) planes of titanium crystals. Notably, in the films crosslinked by the PTCs (including PMTCs, PDTCs, and PPTCs), their diffraction peaks originating from the titanium crystals were observed shifting to higher 2θ angles. This suggests structural changes in the titanium crystals induced by chelation, which have reported been previously [33]. Moreover, the crosslinked films exhibited distinct surface morphologies, as revealed by the SEM images (from Figure 3F to Figure 3M). The neat gelatin film (control) and the PD-, PM-, PP-, and T-crosslinked films displayed smooth and flat surfaces. In contrast, the PMTC-, PDTC-, and PPTC-crosslinked films exhibited numerous particles on their surfaces. These observations, including the variations in diffraction angles and the presence of particles on the film surfaces, provide evidence for the formation of PTCs within the gelatin networks.

### 3.2. Gelatin–PTC Interaction and the Synergistic Crosslinking Effect

**Figure 4:**
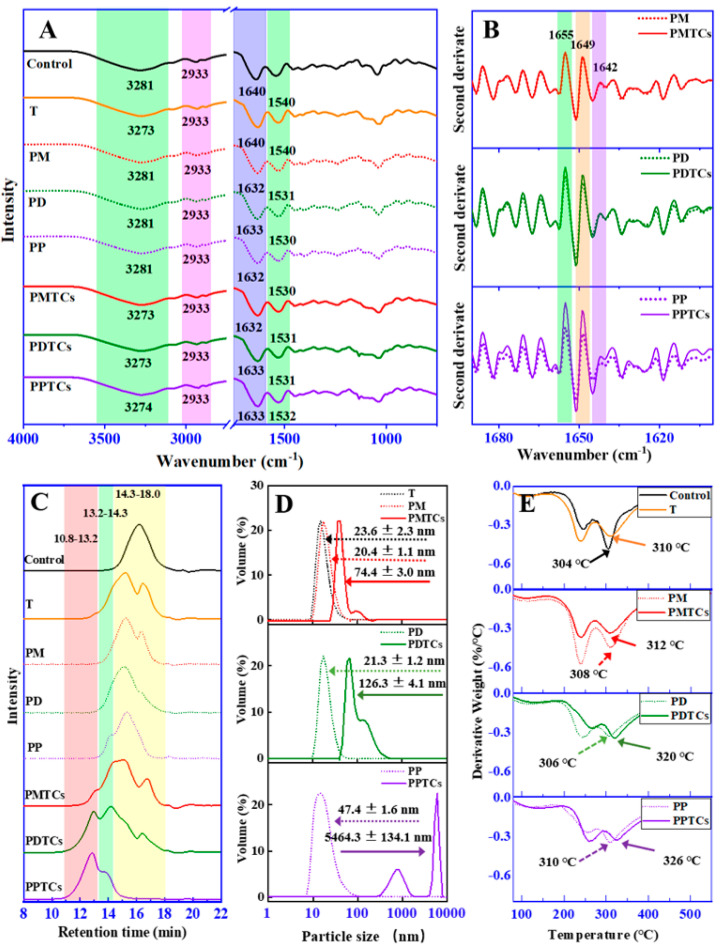
ART-FTIR spectrum (**A**) and second-order-derived spectrum (**B**) of the PTC-crosslinked gelatin films. HPSEC-ELSD chromatogram (**C**) and particle size distribution (**D**) and DTG curve (**E**) of the PTC-crosslinked gelatin.

To gain insights into the underlying mechanisms of the crosslinking, ATR-FTIR spectroscopy were utilized (Figure 4A). For the neat gelatin film (control), absorption bands and peaks were mainly appeared at 3281, 2941, 1640, and 1540 cm^−1^. In accordance with previous reports [21,34], the observed bands and peaks corresponded to different functional groups within the protein structure. Specifically, the peaks appeared at 3281 cm^−1^ was attributed to NH-stretching coupled with hydrogen bonding (Amide-A). While peaks at 2941 cm^−1^ were corresponded to asymmetric C-H stretching (Amide-B). The bands at 1640 cm^−1^ and 1540 cm^−1^, were assigned to the Amide-I and Amide-II, which represents C-O stretching and hydrogen bonding coupled with COO-, NH- bending coupled with CN- stretching, respectively.

After crosslinking by titanium ions or the PTCs, the absorption bands at Amide-A were observed shifting to lower wavenumbers, indicating formation of the coordinate bond between titanium and amide groups on gelatin side chains [21,29]. The Amide-I and Amide-II corresponding peaks were observed shifting to lower values in all the crosslinked films (except T). These results indicated the establishment of hydrogen bonds between polyphenols and the amide and carboxyl groups [11].

The spectral region of 1690–1600 cm^−1^ (Amide-I) was then derivatized to observe the secondary structure of gelatin (Figure 4B). The PD and PDTCs had slightly different heights at 1655, 1649 and 1642 cm^−1^. As for the PP and PPTCs, these height differences were more pronounced. According to Zheng et al. [21], these peaks were associated with different helical structures in gelatin, including triple helix, single α-helix, and disordered helix conformations. The greater the change in gelatin secondary structure, the more obvious the changes that will be observed in these peaks [21]. These findings suggest that PTCs may exert a greater influence on altering gelatin molecular structures compared to polyphenols alone. Additionally, these results imply the possibility of synergistic crosslinking effects attributed to PTCs. Following this hint, crosslinking ability of the PTCs were further explored on the aspect of changes in gelatin hydrodynamic volume, particle sizes and thermal stability.

HPSEC-ELSD analysis revealed that the retention time of neat gelatin (Control) during size exclusion was observed at 14.3–18.0 min (Figure 4C). However, after crosslinking with polyphenols or titanium (PM, PD, PP, and T), this chromatographic peak broadened and shifted to 13.2–14.3 min. These results indicate the aggregation of gelatin molecules induced by crosslinking [35]. When gelatin was crosslinked by PTCs, new peaks with shorter retention times (10.8–13.2 min) were observed, indicating further aggregation of gelatin molecules. Similar trends were observed in the dynamic light scattering (DLS) results (Figure 4D), where the gelatin crosslinked with T, PM, PD, and PP exhibited particle sizes ranging from 20.4 to 47.4 nm. However, after crosslinking with PTCs, the particle sizes further increased to larger values, which can be attributed to crosslinker-induced gelatin aggregation as well [36]. Both HPSEC-ELSD and DLS analyses indicate that gelatin is more prone to aggregation after crosslinking with PTCs compared to crosslinking with polyphenols or titanium alone.

A similar trend appeared for the thermal analysis (Figure 4E). During the heating process, the gelatin films showed three weight loss peaks (50–150 °C, 200–270 °C, and 270–330 °C) on their DTG thermogram. These peaks can be ascribed to the loss of moisture and water molecules associated with gelatin (50–150 °C), thermal degradation of the plasticizer (glycerol), and decomposition of short peptides (200–270 °C), and the thermal denaturation of gelatin networks (270–330 °C) [24,37,38]. The thermal denaturation temperatures of T, PM, PD, and PP were found to be 310 °C, 308 °C, 306 °C, and 310 °C, respectively, surpassing the neat gelatin film (control, 304 °C). This phenomenon suggests that the thermal stability of the gelatin films can be enhanced through crosslinking using polyphenols or titanium, aligning with prior studies [24,39]. More importantly, the highest thermal denaturation temperatures were observed in the PTC-crosslinked samples (PMTCs = 312 °C, PDTCs = 320 °C, and PPTCs = 326 °C), indicating that the highest crosslinking ability were presented by the complexes.

**Figure 5:**
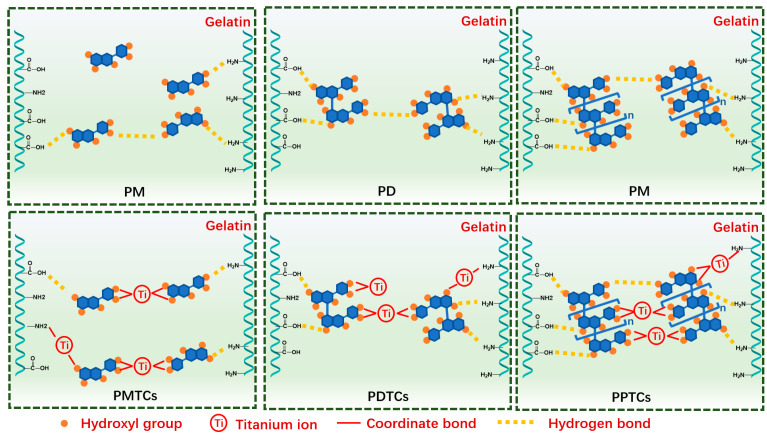
Illustration of synergistic gelatin crosslinking effect provided by PTCs.

In general, the gelatin crosslinked by PTCs demonstrated notable enhancements in hydrodynamic volume, particle sizes, and thermal stability when compared to gelatin crosslinked with titanium or polyphenols alone. These compelling findings strongly suggest that PTCs possess a superior gelatin crosslinking ability in comparison to titanium or polyphenols alone, showcasing a synergistic crosslinking effect. This synergy is attributed to the provision of additional reactive sites by PTCs, which contribute multiple hydrogen and coordinate bonds to reinforce the gelatin networks, as depicted in Figure 5.

Regarding the influence of this synergistic crosslinking effect on practical performances of the film, such as its mechanical behavior, light-blocking capacity, water resistance, and antioxidant potential, an examination and presentation of the findings can be found in the following sections.

### 3.3. Performances of the Gelatin Films Crosslinked by PTCs

#### 3.3.1. Mechanical Properties

**Figure 6:**
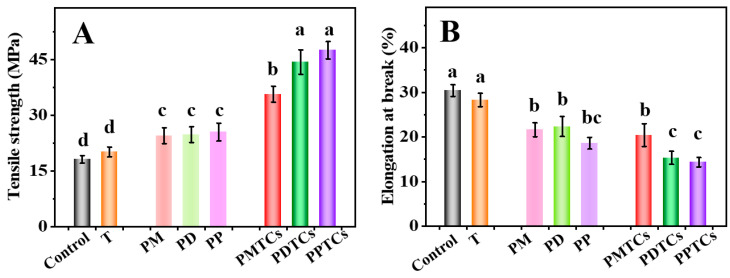
The tensile strength (TS) (**A**) and elongation at break (EAB) (**B**) of the films, different letters above column indicate a significant difference were shown through Tukey’s post-hoc test (*p* < 0.05).

Mechanical characteristics, specifically the TS and EAB, play a pivotal role in the context of biomaterial-based packages [4]. As shown in Figure 6A, the PTC-crosslinked films exhibited remarkable TS values: 30.73 MPa for PMTCs, 44.36 MPa for PDTCs, and 47.55 MPa for PPTCs. These values were 1.7 to 2.6 times higher than that of the neat gelatin film (18.09 MPa) and surpassed the films crosslinked solely by polyphenols or titanium. These findings align with earlier studies that emphasize the beneficial impact of crosslinker interactions with the functional groups within gelatin networks, leading to an improved TS [11,37,40].

Regarding the EAB of the films, the neat gelatin film demonstrated an EAB of 30.4% (Figure 6B). However, films crosslinked solely with titanium or polyphenols exhibited lower EAB values. Notably, the films crosslinked by PTCs displayed the lowest EAB values. The observed pattern in EAB, in contrast to the trend seen in TS, suggests that the crosslinking of PTCs resulted in a decrease in the mobility of gelatin molecules within the film matrix, which ultimately led to a decrease in the film’s flexibility [24,41].

#### 3.3.2. Light Shielding Ability

**Figure 7:**
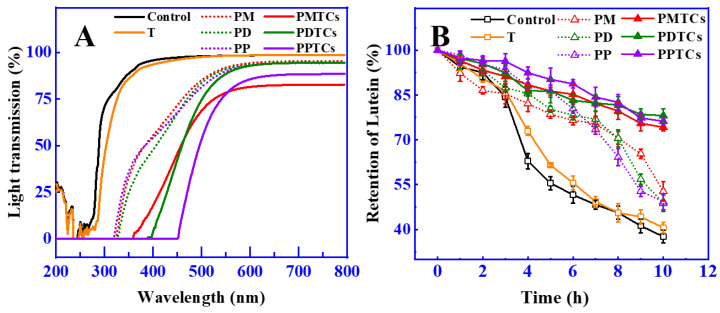
The light transmission property of the films (**A**) and retention of lutein during light irradiation (**B**).

The light shielding characteristics of films can protect food from light induced degradation or spoilage, as many nutritional ingredients are susceptible to light damage [42,43]. The light shielding abilities of PTC-crosslinked films was measured across 200 to 800 nm (including UV and Vis region), as illustrated in Figure 7A. The findings indicated that the polyphenol-crosslinked films demonstrated effective UV-blocking properties, with light transmission rates below 5% in the range of 200 to 320 nm. This observation aligns with previous studies that also demonstrated the increased UV light shielding ability of gelatin films after crosslinking with polyphenols [13,44,45]. The enhanced ability of these films to block light came from the notable absorption of UV light facilitated by the polyphenol molecules [46].

Remarkably, the PTC-crosslinked films demonstrated significant improvements in their light shielding capabilities, as evidenced by the low light transmission rates at 400 nm (PMTCs: 18%, PDTCs: 4%, PPTCs: 1%), which were considerably lower than the light transmission rate of the neat gelatin films (95.75%). These findings align with the observations reported by Zheng et al. [39], where the enhanced light shielding properties were attributed to the structural changes induced by the chemical reactions of the crosslinkers (as shown in Figure 3B). Specifically, in this research, the presence of PTCs resulting from polyphenol–titanium chelation contributed to the expedited absorbance band, further enhancing the light-shielding capabilities [47].

In order to assess the effectiveness of light shielding in preserving food, the retention of lutein was utilized as a measure, since lutein is susceptible to color fading caused by photodegradation [48]. The results showed that (Figure 7B), after 10 h of exposure to a strong light from a 300 W Xe lamp at a distance of 30 cm, the neat gelatin film wrapped around the glass vial retained only 37.6% of the initial lutein content. In contrast, the lutein protected by films crosslinked with polyphenols showed higher levels of retention (PM = 52.8%, PD = 48.8%, and PP = 49.3%) compared to the neat gelatin film. Notably, the films crosslinked by PTCs exhibited superior light barrier properties, resulting in even higher lutein retention (PMTCs = 74.2%, PDTCs = 78.1%, PPTCs = 76.2%). This observed trend in lutein retention paralleled the trend observed in film light shielding, in agreement with Ye’s report [25]. These results highlight the ability of PTC-crosslinked films to provide enhanced food preservation compared to gelatin films, emphasizing their potential in food packaging applications.

#### 3.3.3. Hydrophobicity

The hydrophobicity of the pure gelatin film is insufficient for direct packaging of moisture-rich foods [49]. This limitation arises from its inherent hydrophilic nature [5]. To evaluate the hydrophobicity of the PTC-crosslinked films, measurements of WCA and WS were conducted.

**Figure 8:**
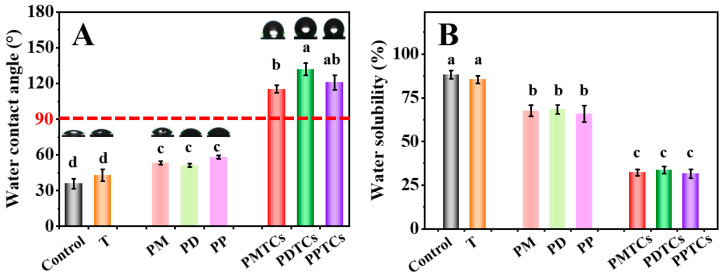
The water contact angle (WCA) (**A**) and water solubility (WS) (**B**) of the films, different letters above column indicate a significant difference were shown through Tukey’s post-hoc test (*p* < 0.05).

As shown on Figure 8A,B, the neat gelatin film (control) was shown to have a hydrophilic surface (WCA = 35.8°) and prone to dissolve by water (WS = 88.2%), and can be consistent with the previous findings [38,50]. Upon crosslinking with polyphenols, WCA of the films were improved (PM = 53.3°, PD = 51.2°, PP = 58.1°). This improvement can be attributed to the hydrophobicity induced by the exposed benzyl rings on polyphenols, as the hydroxyl groups of polyphenols are occupied by the gelatin side chains for crosslinking [51]. In contrast, films crosslinked with PTCs exhibited significantly higher WCA (PMTCs = 115.3°, PDTCs = 131.9°, PPTCs = 120.9°), surpassing the threshold point of hydrophobicity (>90°, hydrophobic). Similarly, the polyphenols crosslinked films showed lower WS than neat gelatin film, while the PTC-crosslinked film showed lowest WS values (PMTCs = 32.1%, PDTCs = 33.6%, PPTCs = 31.5%). As for the water swelling ratios and the water vapor transmission rates of the films, as well as the stabilities of the films in acidic, base, NaCl solutions showed similar trends (Tables S3 and S4).

Referring to earlier studies, the enhancement in film hydrophobicity can be ascribed to the intrinsic hydrophobic characteristics of the PTCs [23,52], thereby rendering them more appropriate for packaging food items that have high water content.

#### 3.3.4. Antioxidant Abilities

A favorable strategy for improving the antioxidant function of packaging films is the integration of polyphenols into the matrix [53]. Nevertheless, a crucial obstacle that requires attention is the regulated and extended release of these antioxidant compounds [54]. To address this issue, the radical scavenging ability of the films and the release of polyphenols from the films were assessed.

Table 2 indicates a significant enhancement in the antioxidant activities of gelatin films, including DPPH, ABTS, and FRAP scavenging abilities, following PTC crosslinking. These improvements can be attributed to the inherent phenolic hydroxyl groups in polyphenols, providing notable DPPH, ABTS, and FRAP radical scavenging capabilities [6]. Furthermore, the antioxidant activity after PTC crosslinking exceeds that of the films crosslinked solely with polyphenols. According to the previous research [53], this phenomenon is attributed to the coordination-induced extension of the polyphenol molecule structure, facilitating the exposure of phenolic hydroxyl groups in polyphenols.

**Figure 9:**
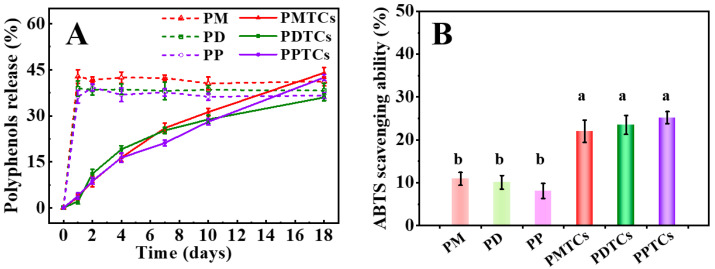
The polyphenol release in the film-immersed water (**A**) and ABTS scavenging ability after 18 days of water immersion (**B**), different letters above column indicate a significant difference were shown through Tukey’s post-hoc test (*p* < 0.05).

In Figure 9A,B, it can be observed that films crosslinked with PTCs demonstrated a gradual release of polyphenols during the 18-day water immersion period. At the 18th day of water immersion, the PTC-crosslinked films exhibited ABTS radical scavenging abilities of 22.0% (PMTCs), 23.5% (PDTCs), and 25.2% (PPTCs), which were nearly twice as high as the corresponding films crosslinked with polyphenols alone. In contrast, the films crosslinked with polyphenols exhibited an initial release of 40% of polyphenols after 1 day of water immersion, and thereafter the concentration of polyphenols in the immersed water remained constant. Furthermore, the water used for immersion of the polyphenol crosslinked films showed significantly lower ABTS scavenging abilities compared to the immersion water from PTC-crosslinked films.

The delayed release of polyphenols from PTC-crosslinked films can be attributed to their lowered WS values [11]. This character contributes to their superior antioxidant ability, as it delays the oxidation of polyphenols [25]. This is similar to the report from Soltanzadeh [10], who observed that increasing the hydrophobicity of films can prolong the migration and release of incorporated antioxidants. These results highlight that the incorporation of PTCs may enhance the efficacy of films in preserving food products and providing sustained antioxidant protection.

In summary, the application of Polyphenol Tannin Complexes (PTCs) in crosslinking treatments significantly improves the physical and mechanical attributes, light-blocking efficacy, hydrophobicity, and antioxidant qualities of gelatin films. Notably, films crosslinked with PTCs exhibit a tensile strength surpassing those crosslinked with alternative plant phenols or extracts abundant in polyphenols, including epigallocatechin gallate [55], green tea extracts, and grape seed extracts [6]. The hydrophobic surface characteristics, as measured by the contact angle, also surpass those of the films crosslinked with other crosslinkers, such as polyphenols [39], proteins [21], and polysaccharides [10]. Additionally, hop tannins assume a crucial role as additives for flavor modulation in beer and are readily accessible as commercially available products, making them more convenient compared to other plant extracts. Moreover, the biological toxicity of titanium sulfate (LD50 = 2140 mg/kg, SDS No. E8209E-2) is notably lower than that of the primary crosslinking agent currently employed in edible film production, glutaraldehyde (LD50 = 77 mg/kg, SDS No. 3437E-3) [56]. Therefore, the use of PTCs is a feasible choice.

## 4. Conclusions

In this study, we investigated the coordination of polyphenols (monomers, dimers, and polymers) with titanium within gelatin films, resulting in the formation of PTCs as crosslinkers. The PTCs demonstrated synergistic crosslinking effects through the establishment of multiple hydrogen and coordinate bonds within the gelatin network. As a result, the gelatin films crosslinked with PTCs exhibited significant improvements compared to films crosslinked solely with polyphenols or titanium. The PTC-crosslinked films exhibited significant improvements in their mechanical properties, demonstrating a remarkable tensile strength of up to 47.55 MPa, which is 2.6 times greater than that of the pristine gelatin film. Additionally, these films demonstrated effective protection against UV light within the 200 to 400 nm range, with minimal light transmission of up to 1%. This enhanced light shielding capability contributed to a lutein retention rate of up to 78.1%. Furthermore, the integration of PTCs resulted in enhanced film hydrophobicity, as indicated by water contact angles surpassing 131.9° and a water solubility that is lower than 33.6%. Moreover, the films crosslinked with PTCs showcased a prolonged release of polyphenols, lasting for as long as 18 days, accompanied by a commendable free radical scavenging capacity of up to 25.2%. These improved attributes of the gelatin films, facilitated by the presence of PTCs, bestow them with exceptional functionality across multiple domains, rendering them highly suitable for a wide range of food packaging applications.

## Figures and Tables

**Table 1:** Composition of the gelatin blends prepared for film casting (wt% of gelatin).

Sample	Polyphenol	Ti(SO_4_)_2_	Glycerol
Control	0	0	25
T	0	10	25
PM	10	0	25
PD	10	0	25
PP	10	0	25
PMTCs	5	5	25
PDTCs	5	5	25
PPTCs	5	5	25

**Table 2:** Radical scavenging abilities of the films (Trolox equivalents, μM Trolox/ g film) *.

	DPPH	ABTS	FRAP
PMTCs	8.21 ± 1.02 ^a^	9.72 ± 1.26 ^a^	3.61 ± 0.42 ^a^
PDTCs	8.37 ± 1.32 ^a^	8.31 ± 1.02 ^a^	3.42 ± 0.41 ^a^
PPTCs	7.46 ± 0.55 ^a^	7.31 ± 0.64 ^a^	2.83 ± 0.36 ^a^
PM	4.62 ± 0.87 ^b^	4.66 ± 0.41 ^b^	1.62 ± 0.23 ^b^
PD	4.33 ± 1.21 ^b^	5.32 ± 0.46 ^b^	1.53 ± 0.18 ^b^
PP	4.31 ± 0.64 ^b^	4.37 ± 0.73 ^b^	1.72 ± 0.24 ^b^
Control	0.0 ± 0.02 ^c^	0.67 ± 0.42 ^c^	0.09 ± 0.07 ^c^

* Different letters within a row indicate a significant difference were shown through Tukey’s post-hoc test (*p* < 0.05).

## Data Availability

All data generated or analyzed during this study are included in this published article and its Supplementary Materials.

## Supplementary Materials

The following supporting information can be downloaded at: https://www.mdpi.com/article/10.3390/antiox13020167/s1, Protocol S1: Protocol applied for proanthocyanidin polymer extraction and purification; Figure S1: Method used for ^13^CNMR analysis, and the results obtained from the analysis; Figure S2: The TIC chromatography and MS/MS spectrum of the subunits from proanthocyanidin polymers (hops tannin); Figure S3: TGA results obtained from the crosslinked films; Figure S4: UV-Vis spectrum and fluorescence spectrum of the PTCs composed of different polyphenol–titanium ratios; Table S1: Composition of subunits from proanthocyanidin polymers (hops tannin); Table S2: Average thickness of the films; Table S3: The swelling ratio, impact of pH, temperature, and ionic strength of the films; Table S4: The water vapor transmission rates of the films. References [57,58,59,60] are cited in the supplementary materials.
