# Supplementary material for: A High-Performance Food Package Material Prepared by the Synergistic Crosslinking of Gelatin with Polyphenol–Titanium Complexes
Source: Antioxidants (Basel). 2024 Jan 29;13(2):167. doi: 10.3390/antiox13020167 (PMC10885897; doi:10.3390/antiox13020167)
Supplement: Supplementary file 1 [file antioxidants-13-00167-s001.zip › antioxidants-2768800-supplementary.pdf]

Supplementary Information

A High-Performance Food Package Material Prepared by Synergistic Crosslinking of Gelatin with Polyphenol-Titanium Complexes

Wanqin Zhang[1], Jiaman Liu[1], Tao Zhang [2]*, and Bo Teng [1,3]*

1 College of Science, Shantou University, Shantou 515063, China;

2 Key Laboratory of Marine Materials and Related Technologies, Ningbo Institute of Materials Technology and Engineering, Chinese Academy of Sciences, Ningbo 315201, China;

3 Guangdong Provincial Key Laboratory of Marine Biotechnology, Shantou University, Shantou 515063, China;

* Correspondence: tzhang@nimte.ac.cn (T.Z.); bteng@stu.edu.cn (B.T.)

Protocol S1. The protocol applied for proanthocyanidin polymer extraction and purification

Extraction and purification of the proanthocyanidin polymers were proceeded as described in our previous research[1] and provided as following. A 200g fresh hops were ground into fine powders, then transferred into an acetone–water solution (4.0 L, acetone: water= 7: 3, v: v). The extraction was provided at 25°C for 48h. Afterwards, the residue, acetone and lipids were removed by a paper filtration, a rotary evaporation (reduced pressure, 35 °C) and dichloromethane (1 L, 25 °C for 12 h) extraction. Then, the crude proanthocyanidin polymers were obtained after a rotary evaporation (reduced pressure, 35 °C) followed by a lyophilization. 1.0 g of crude polymers were dissolved in 50% methanol solution (10 mL) then loaded onto a Sephadex LH-20 column (column bed volume = 800 mL). The column was washed with 5.0 L methanolic solution (50%) with a 25 mL/min flow rate to remove simple phenolics, proteins, and sugars. Proanthocyanidin polymers were then collected by elution with 1.0 L acetone solution (70%). Afterwards, purified proanthocyanidin polymers were obtained after a rotary evaporation (35 °C) and a lyophilization. A methyl cellulose precipitation assay was taken and referenced with Kennedy's report[2], the result showed purity of the obtained proanthocyanidin polymers was 94.3 ± 1.2%.

Figure S1. Method used for ^{13}CNMR analysis and the results obtained from the analysis

^{13}CNMR spectra of the purified hop tannins indicated that these tannins were composed of flavon-3-ol subunits. The spectra were obtained with a Bruker Ascend 400 MHz NMR spectrometer (Bruker BioSpin, Rheinstetten, Karlsruhe, Germany), while 30 mg tannins were dissolved in a 750 μL CD_4O-D_2O solution (1:1, V:V).

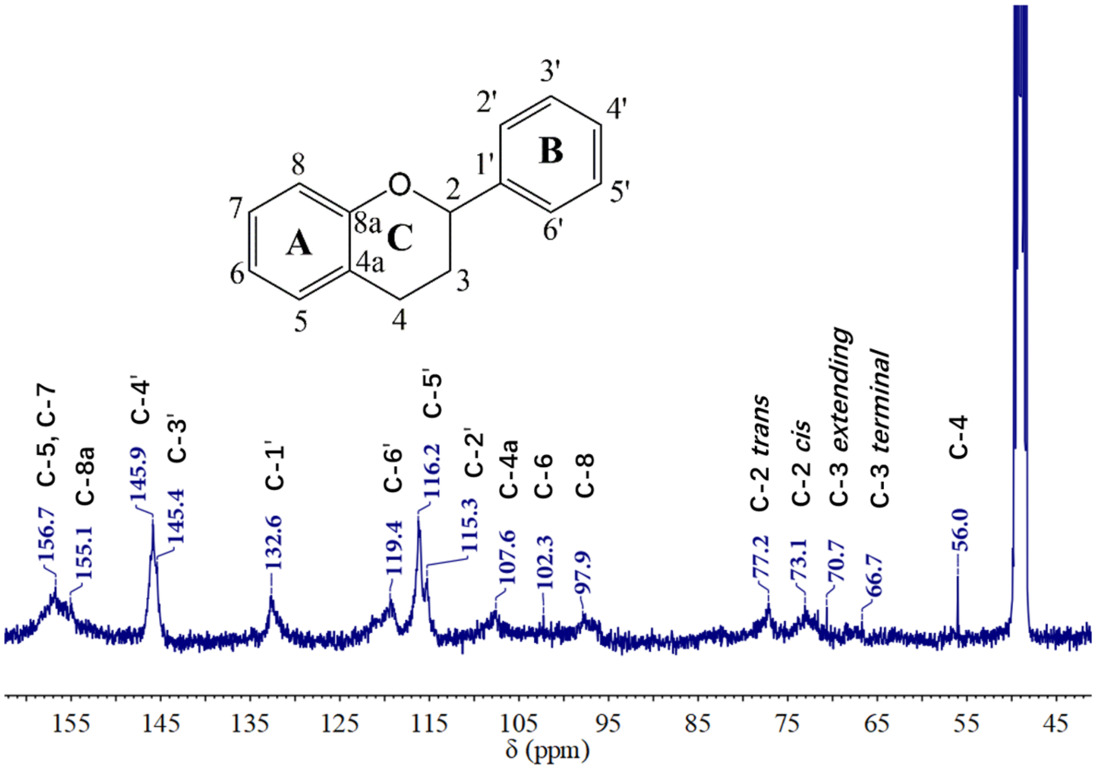

Signals on NMR spectrum (Figure S1) were assinged referrecing with a previous study [3] and corresponding carbons were presented. These results were reprinted (adapted) with permission from Jiaman Liu, Yanbiao Chen, Xinxin Zhang, Jie Zheng, Weiying Hu and Bo Teng, 2022. Hop Tannins as Multifunctional Tyrosinase Inhibitor: Structure Characterization, Inhibition Activity, and Mechanism. Antioxidants, 2022, 11, 772.

Figure S2. The TIC chromatography, MS/MS spectrum and composition of the subunits from proanthocyanidin polymers (hops tannin)

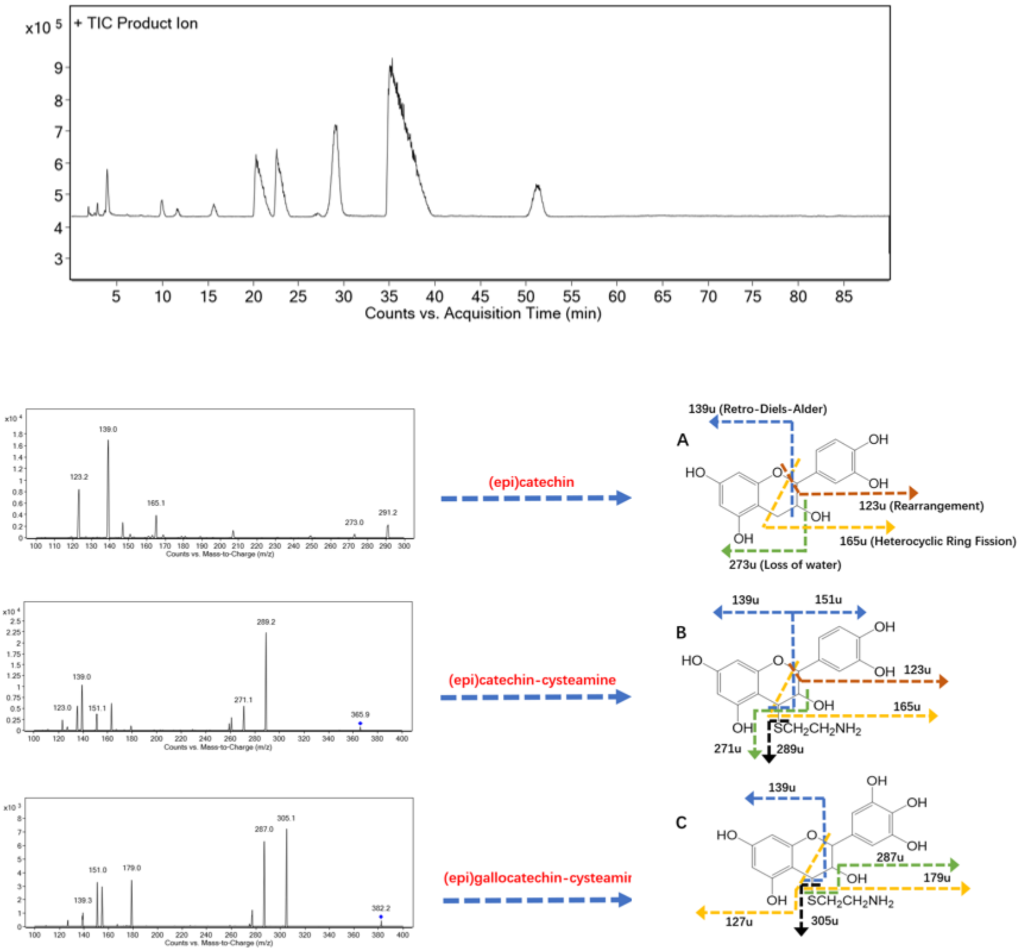

The proanthocyanidin polymers were treated with acid–cleavage derivatization treatment prior to analysis. The derivatization was carried out referenced with our previous report[4]. In short, 20 mg of tannins was dissolved in 200 μL of cysteamine hydrochloride solution (50 mg/L, dissolved in methanol); then, 50 μL of 32% hydrochloride was added and heated in a 65 ˚C water bath (for 30 min). Afterwards, 1250 μL of sodium acetate solution (140 mM) was added to stop the derivatization. 10 μL sample aliquot was injected into a Synergi Hydro-RP column (150 mm × 2 mm, 4 μ, 80 Å), which was mounted onto the HPLC–ESI–MS/MS instrument (Agilent 1290II-6460, California, CA, USA). The column was eluted with mobile phase A and B composed of formic acid/water/acetonitrile (phase A = 0.3: 99.7: 0, phase B = 0.3: 59.7: 40, v: v: v). A linear gradient elution (from 0 to 90 min, 6–20% B) was used with a 0.3 mL/min flow rate. Elutes from HPLC were analyzed under the multiple-reaction monitoring (MRM) mode and recorded from

m/z 100 to 500.

Based on the peak areas from each compound shown on the TIC and the corresponding MS/MS spectrum (Figure S2), the subunits were quantified, and the average polymerization degree was subsequently calculated (Table S1) in accordance with Kennedy's report [5]. These results were reprinted (adapted) with permission from Jiaman Liu, Yanbiao Chen, Xinxin Zhang, Jie Zheng, Weiying Hu and Bo Teng, 2022. Hop Tannins as Multifunctional Tyrosinase Inhibitor: Structure Characterization, Inhibition Activity, and Mechanism. Antioxidants, 2022, 11, 772.

Figure S3. TGA results obtained from the crosslinked films.

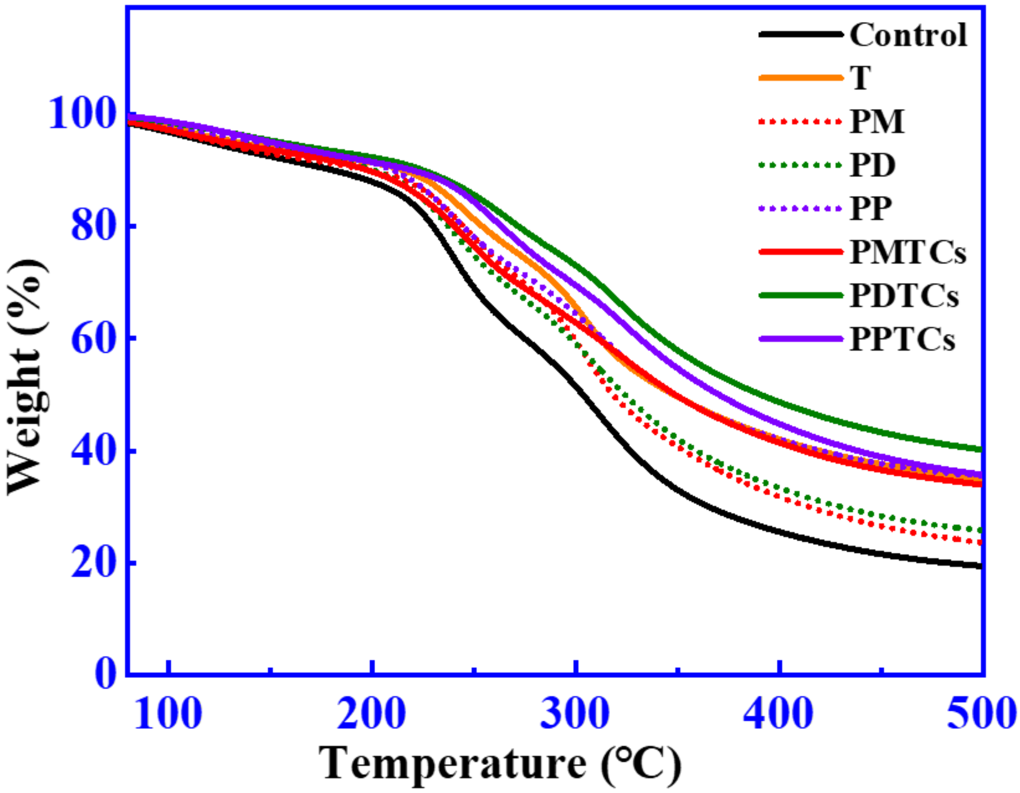

Figure S4. UV-Vis spectrum and fluorescence spectrum of the PTCs composed of different polyphenol-titanium ratios.

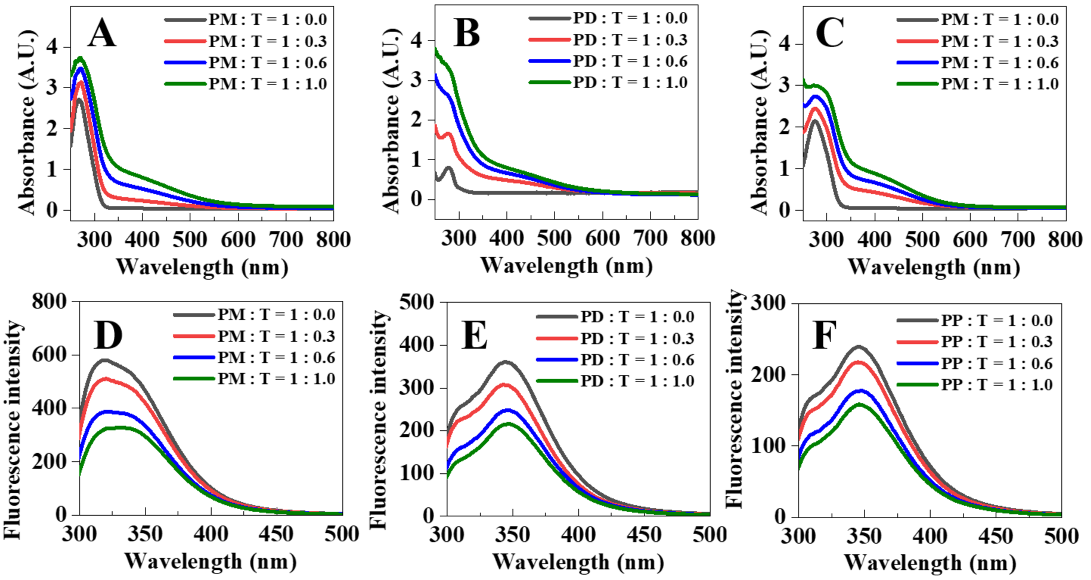

The UV-vis spectrum (A to C) and the fluorescence spectrum (D to E) of the PTCs composed of constant polyphenol concentration and varied titanium concentration. The results were obtained using the following procedure: A 1 mL aliquot of the polyphenol solution (0.125 mg/mL) was taken and added to various centrifuge tubes. Subsequently, these were individually mixed with 0.0 mL, 0.3 mL, 0.6 mL, and 1.0 mL of the Ti(SO4)2 solution (0.125 mg/mL). Finally, distilled water (1.0 mL, 0.7 mL, 0.4 mL, and 0.0 mL) was added to the respective tubes to achieve a final sample volume of 2.0 mL. The UV-Vis and fluorescence spectrum were taken as described in part 2.2.2 and 2.2.3 in the main text.

Table S1. Structural information of the polyphenol obtained from the HPLC-ESI-MS/MS and the structure of polyphenol monomer and dimer used in current research[1]

	Terminal units	Extensional units	(Average) Polymerization degree
Monomer (catechin)	catechin	-	1
Dimer (procyanidin B_2)	epicatechin	epicatechin	2
Polymers (obtained from hops) [1]	(epi)catechin	(epi)catechin, (epi)gallocatechin	10.2

[1] Structural information of the polyphenol polymer (hop tannins) was obtained from our previous research[1].

Table S2. Average thickness of the films.

Crosslinker	Thickness (mm)
Control	0.55 ± 0.12 [a]
T	0.51 ± 0.13 [a]
PM	0.52 ± 0.13 [a]
PD	0.53 ± 0.12 [a]
PP	0.51 ± 0.11 [a]
PMTCs	0.53 ± 0.12 [a]
PDTCs	0.52 ± 0.11 [a]
PPTCs	0.53 ± 0.10 [a]

The average thickness of the films, determined by measuring the thickness at 12 random locations using a spiral micrometer.

Table S3 The swelling ratio and remaining mass of the films after 5h immersion in acidic (pH = 3.6), base (pH = 10.0) and NaCl (1M) solutions

	**Swelling ratio1 (%)**	**Remaining mass of the films after 5h immersion in acidic, base and NaCl solutions**		
		**Acidic solution (pH = 3.6)**	**Base solution (pH = 10.0)**	**NaCl solution (1M) solutions**
PMTCs	20.4 ± 7.6b	82.1 ± 4.2a	95.4 ± 2.6a	96.3 ± 4.7a
PDTCs	23.0 ± 2.3b	83.6 ± 6.3a	96.3 ± 3.1a	95.2 ± 4.2a
PPTCs	21.4 ± 8.4b	84.3 ± 3.7a	96.6 ± 3.6a	91.4 ± 4.6a
PM	47.6 ± 3.1a	57.2 ± 2.1b	66.2 ± 2.8b	63.5 ± 4.3b
PD	52.3 ± 8.2a	56.4 ± 5.2b	65.1 ± 2.9b	66.2 ± 4.1b
PP	56.1 ± 6.3a	52.1 ± 6.1b	63.5 ± 4.5b	66.6 ± 4.4b

The procedure for assessing the swelling ratio of gelatin films was as follows: A 10g gelatin film was submerged in 500 mL of water within a flask and then positioned in a water bath at 30°C, where it was agitated at 60 rpm for a duration of 12 hours. Following the completion of the immersion period, the gelatin film was retrieved, and the average film thickness was determined

by measuring from 12 random positions and subsequently calculating the mean. The swelling ratio (%) was then computed by dividing the change in the average thickness of the film before and after immersion by the initial thickness of the film.

The stability of gelatin films in acidic, alkaline, and NaCl solutions was evaluated by measuring the remaining mass of the gelatin films after 5 hours of immersion in the following solutions: 1) NaH2PO4 buffer solution (0.5M, pH = 3.6); 2) Sodium carbonate-sodium bicarbonate buffer solution (0.1M, pH = 10.0); 3) NaCl solution (1M), respectively. For each analysis, 10g of gelatin film was submerged in 500 mL of the corresponding solution. Subsequently, the flasks were placed on a water bath shaker at 30°C for 5 hours. Following agitation, the gelatin films were extracted, and any excess solution was drained before drying in an oven (120°C) until a constant weight was attained. The residual mass was then calculated by determining the change in weight before and after immersion, divided by the initial thickness of the gelatin film.

Table S4 The water vapor transmission rate of the films

Sample	WVTR (g/mm/m^2 d atm @ 50 % rh)
PMTCs	810 ±61b
PDTCs	731 ± 17b
PPTCs	850 ± 61b
PM	1312 ± 11a
PD	1276 ± 14a
PP	1347 ± 96a

The water vapor transmission rate (WVTR) of the films were assessed following the ASTM standard method (ASTM 1987. Standard methods for water vapor transmission of materials (E 96–80). Annual Book of ASTM Standards. Philadelphia, PA: American Society for Testing and Materials). Each gelatin film, cut into 90 × 90 mm pieces, was positioned on a permeability cup previously loaded with fused calcium chloride. After sealing the cup with a cover, it was placed in a humidity chamber maintained at 25°C and 50% relative humidity (RH) for 24 hours. The weight of the sealed cup was recorded at the start and at one-hour intervals over the entire testing duration.

Reference

1. Liu, J.; Chen, Y.; Zhang, X.; Zheng, J.; Hu, W.; Teng, B. Hop Tannins as Multifunctional Tyrosinase Inhibitor: Structure Characterization, Inhibition Activity, and Mechanism. Antioxidants 2022, 11, 772.
2. Sarneckis, C.J.; Dambergs, R.G.; Jones, P.; Mercurio, M.; Herderich, M.J.; Smith, P.A. Quantification of condensed tannins by precipitation with methyl cellulose: development and validation of an optimised tool for grape and wine analysis. Aust. J. Grape Wine Res. 2006, 12, 39-49, doi:10.1111/j.1755-0238.2006.tb00042.x.
3. Davis, A.L.; Cai, Y.; Davies, A.P.; Lewis, J.R. 1H and 13C NMR Assignments of Some Green Tea Polyphenols. Magnetic Resonance in Chemistry 1996, 34, 887-890, doi:https://doi.org/10.1002/(SICI)1097-458X(199611)34:11<887::AID-OMR995>3.0.CO;2-U.
4. Teng, B.; Hayasaka, Y.; Smith, P.A.; Bindon, K.A. Precipitation of Tannin-Anthocyanin Derivatives in Wine is Influenced by Acetaldehyde Concentration and Tannin Molecular Mass with Implications for the Development of Nonbleachable Pigments. Journal of Agricultural and Food Chemistry 2021, 69, 4804-4815, doi:10.1021/acs.jafc.1c00396.
5. Kennedy, J.A.; Taylor, A.W. Analysis of proanthocyanidins by high-performance gel permeation chromatography. J. Chromatogr. A 2003, 995, 99-107, doi:10.1016/s0021-9673(03)0042.
